# Effectiveness of Atrial Natriuretic Peptide in the Treatment of Critically Ill Patients: A Systematic Review and Meta-Analysis

**DOI:** 10.3390/jcm14103267

**Published:** 2025-05-08

**Authors:** Peter Olujimi Odutola, Ayodeji Olarewaju, Priyank Shah

**Affiliations:** 1 Department of Molecular and Cellular Biology, Harvard University, Massachusetts Hall, Cambridge, MA 02138, USA; 2 Medical College of Georgia SW Campus, Albany, GA 31701, USA; aolarewa@phoebehealth.com; 3 Novant Health Heart and Vascular Institute, Charlotte, NC 28204, USA; priyank_221084@yahoo.com

**Keywords:** atrial natriuretic peptide (ANP), critically ill patients, systematic review, meta-analysis, renal function

## Abstract

**Background**: Atrial natriuretic peptide (ANP) has emerged as a potential therapeutic agent in critical care settings due to its physiological effects on diuresis, natriuresis, and vasodilation. Despite several promising preclinical data, their clinical utility remains controversial, necessitating a comprehensive evaluation of existing evidence. **Methods**: A systematic review and meta-analysis were conducted following PRISMA guidelines. Searches were performed in PubMed, Google Scholar, and Cochrane databases. Fifteen studies (*n* = 7187) comparing ANP to placebo in critically ill patients were included. Primary outcomes included mortality, hospital length of stay, ICU length of stay, and serum creatinine level. Risk ratios and mean differences with 95% confidence intervals were calculated using random-effects models. **Results**: ANP therapy showed no significant impact on mortality (RR 1.03, 95% CI: 0.89–1.19, *p* = 0.72) but significantly reduced hospital length of stay (MD −1.81 days, 95% CI: −1.91 to −1.72, *p* < 0.00001). ICU length of stay showed no significant difference between groups in subgroup analysis (MD +0.10 days, 95% CI: −0.03 to 0.23, *p* = 0.15). Subgroup analysis revealed improved creatinine levels with ANP (MD −0.19, 95% CI: −0.20 to −0.19, *p* < 0.00001), though high heterogeneity was noted across outcomes. **Conclusions**: ANP therapy shows promise in shortening hospital stays and enhancing renal function in select patients, but its effectiveness varies widely across clinical settings. Large-scale, multicenter studies are necessary to determine the ideal patient groups for ANP therapy in critical care.

## 1. Introduction

Natriuretic peptides, including atrial natriuretic peptide (ANP) and brain natriuretic peptide (BNP), have gained recognition as promising therapeutic agents due to their diverse physiological effects. These peptides promote diuresis, natriuresis, vasodilation, and inhibition of the renin–angiotensin–aldosterone system (RAAS). Research suggests they may enhance cardiac function, improve renal blood flow, and help maintain fluid balance in critically ill patients [1,2].

Despite notable advancements, the management of critically ill medical and surgical cardiac patients remains a significant global challenge, contributing substantially to morbidity and mortality [3,4]. Critical illness, characterized by life-threatening dysfunction of vital organs, affects millions of individuals on an annual basis, with mortality rates in intensive care units fluctuating between 10% and 30%. Prevalent complications, including fluid overload and acute kidney injury (AKI), significantly exacerbate adverse outcomes and prolong hospital admissions [5]. The ramifications of critical illness extend beyond the affected patients and their families, imposing considerable stress on global healthcare systems, thereby underscoring the necessity for the investigation of innovative therapeutic approaches. Natriuretic peptides emerged as potential therapeutic agents for critically ill cardiac patients in the early 1980s [6]. First identified in 1981 as a cardiac-derived peptide integral to the regulation of volume status, atrial natriuretic peptide (ANP) quickly garnered significant scientific interest as a potential therapeutic agent in critical care medicine. Preclinical studies demonstrated its pronounced natriuretic, diuretic, and vasodilatory effects, providing a strong foundation for its clinical evaluation in a range of pathological conditions, including heart failure, renal dysfunction following cardiac surgery, and septic shock [7].

ANP has been extensively studied in the context of heart failure, cardiac surgery, and AKI. Its physiological effects are mediated through cyclic guanosine monophosphate (cGMP)-dependent pathways, which enhance the glomerular filtration rate (GFR) and improve renal function [6]. Clinical trials and observational studies have highlighted ANP’s potential to mitigate complications associated with cardiopulmonary bypass, reduce postoperative renal dysfunction, and improve fluid management strategies [8,9]. However, the broader adoption of natriuretic peptides as therapeutic agents in critical care has been limited due to inconsistent outcomes in randomized controlled trials (RCTs) and concerns about adverse effects, such as dose-related hypotension [10].

Although low-dose ANP infusions have demonstrated promising results in specific patient groups, evidence supporting their routine use in critically ill populations remains inconclusive [11]. Although early findings were promising, clinical trials have yielded mixed results; while some studies reported enhancements in renal function and fluid management, others failed to demonstrate reductions in mortality or morbidity. Additionally, concerns over adverse effects—most notably hypotension at higher doses—have hindered the widespread incorporation of ANP into standard ICU practice [12]. Prospective trials consistently underscored the need for well-designed, large-scale RCTs to better define the therapeutic role of natriuretic peptides in critical care.

This systematic review and meta-analysis aim to evaluate the current evidence regarding the effectiveness of natriuretic peptides in critically ill patients. By synthesizing data from existing clinical studies, this review seeks to update the knowledge base regarding the therapeutic utility of natriuretic peptides and their potential harm in critically ill patients.

## 2. Methods

### 2.1. Eligibility Criteria

Studies that met the following eligibility criteria were included in this meta-analysis:

1. Randomized trials or observational cohorts involving critically ill patients, who are defined as those admitted to intensive care units (ICUs), high-dependency units (HDUs), or presenting with life-threatening conditions such as acute kidney injury, cardiogenic shock, or postoperative complications requiring intensive monitoring and organ support.
2. Studies comparing atrial natriuretic peptide to placebo.
3. Studies that reported results which focused on our objectives.
4. Studies that were available for full review.
5. Studies published in English.

Exclusion criteria:

1. Studies without a control group.
2. Studies with patients overlapping populations.
3. Case series or case reports.
4. Previous systematic reviews and meta-analysis.

### 2.2. Search Strategy and Screening

**Figure 1:**
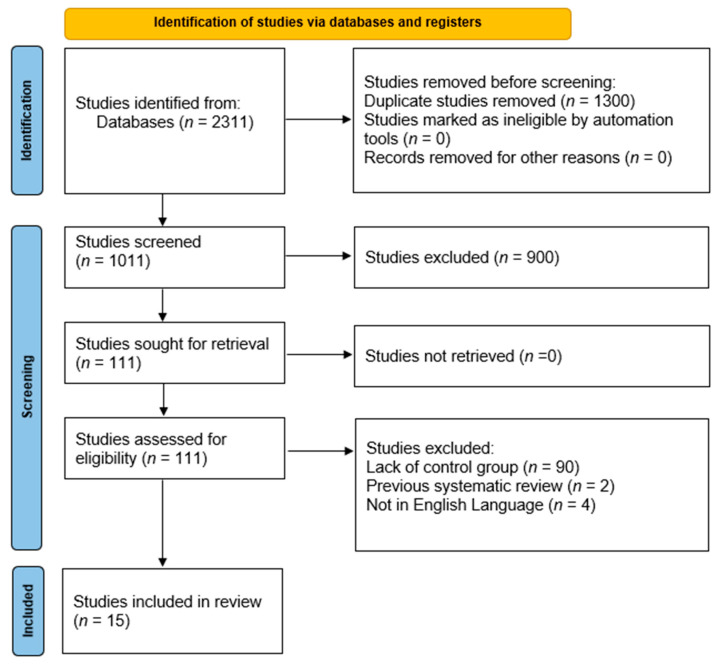
PRISMA flow chart.

A systematic database search was conducted in January 2025 in compliance with the Preferred Reporting Items for Systematic Reviews and Meta-Analyses (PRISMA) guidelines, with no restrictions on publication dates Figure 1 [13]. Even though the systematic review was performed according to PRISMA guidelines, it was not registered as it is not mandatory to do so. We searched PubMed, Google Scholar, and Cochrane Review to find relevant studies. The following search phrases were used to search for papers that included them in their title or abstract: (“Natriuretic Peptides”[Mesh] OR “Natriuretic Peptide, Brain”[Mesh] OR “natriuretic peptide*” OR “brain natriuretic peptide*” OR “B-type natriuretic peptide*” OR BNP OR “NT-proBNP” OR “N-terminal pro-brain natriuretic peptide*” OR “N-terminal pro-B-type natriuretic peptide*” OR “atrial natriuretic peptide*” OR “A-type natriuretic peptide*” OR ANP OR “ProANP” OR “Pro-atrial natriuretic peptide*” OR “C-type natriuretic peptide*” OR CNP) AND (“Critical Illness”[Mesh] OR “Intensive Care Units”[Mesh] OR “Critical Care”[Mesh] OR “critic* ill*” OR “critical* sick*” OR “severe* ill*” OR “acute* ill*” OR “life threat* ill*” OR “critical condition*” OR “ICU patient*” OR “intensive care unit*” OR “critical care” OR “high dependency unit*” OR “HDU patient*” OR “emergency care patient*” OR “acute care patient*” OR “life support patient*” OR “ventilat* patient*” OR “seriously ill” OR “gravely ill” OR “acutely unwell”) AND (“Therapeutics”[Mesh] OR “therapeutic use”[Subheading] OR therap* OR treat* OR intervention* OR administration OR management OR “drug therapy” OR infusion* OR medication* OR “therapeutic use*” OR “clinical use*” OR prescri* OR administ* OR dos*). Only texts published in English were included, with no restrictions on the age of the participants. Two authors/reviewers (AO and PS) independently reassessed the selected manuscripts for inclusion, evidence grading, and data extraction. Any discrepancies in the data were resolved by a third reviewer (PO). Additionally, related articles that surfaced during the review process were also evaluated. In addition to database searching, the reference lists of relevant systematic reviews and meta-analyses were manually screened to identify any additional eligible studies that may not have been captured during the initial search. This step was undertaken to ensure comprehensive study identification and to minimize the risk of omitting relevant citations. This review was not registered in a prospective database such as PROSPERO. The authors acknowledge that protocol registration is increasingly encouraged to enhance transparency and reduce duplication; however, given the scope and nature of this unfunded, retrospective analysis of published data, we proceeded without protocol registration.

### 2.3. Endpoints of the Systematic Review

The primary outcomes were in-hospital mortality, hospital length of stay, intensive care unit (ICU) length of stay, and serum creatinine level on the day of discharge. The secondary objective was blood urea nitrogen level on discharge.

### 2.4. Data Extraction

Each manuscript was independently assessed for inclusion and data extraction by two reviewers. Relevant information for this meta-analysis included the author, year of publication, and key outcomes such as mortality, length of hospital stay, length of ICU stay, serum creatinine levels, and blood urea nitrogen levels. Data extraction was performed using a standardized form: one author extracted the data from the articles, while the other verified its accuracy. Study authors were not contacted for additional data. This decision was made because the required outcome data were sufficiently reported in the published articles. While we recognize that contacting authors is recommended in some review guidelines, feasibility limitations (e.g., older studies and restricted contact access) led us to proceed with analysis based on published information only.

### 2.5. Quality Assessment

The quality of the included randomized controlled trials (RCTs) was assessed using Cochrane’s method for evaluating bias in randomized trials. This approach categorizes studies into five domains: selection, performance, detection, attrition, and reporting—and rates each as having a high, low, or unclear risk of bias. Observational studies were evaluated using the Newcastle–Ottawa Scale (NOS), which assesses the quality of participant selection, comparability of groups, and measurement of exposure.

### 2.6. Statistical Analysis

The data obtained were analyzed using Review Manager V5.4.1 (Cochrane, London, UK). Heterogeneity was assessed using Higgins’ I^2^, and the studies were analyzed using a random-effects model. Continuous data were evaluated using the mean difference, while dichotomous data were expressed as risk ratios (RRs) with 95% confidence intervals (CIs). Results with *p*-values less than 0.05 were considered statistically significant. For studies with significant heterogeneity (I^2^ > 50%), a subgroup analysis was explored to find the possible causes.

### 2.7. Subgroup Analysis

Predefined subgroup analyses were conducted to explore potential sources of heterogeneity across included studies. Specifically, subgroup analyses were planned for the following outcomes:

- Hospital length of stay;
- ICU length of stay;
- Serum creatinine level at discharge.

These subgroup analyses were limited to studies that explicitly reported the relevant outcome with comparable metrics. Subgroups were selected based on outcome categories, not patient-level clinical characteristics.

## 3. Results

A total of fifteen studies satisfied the inclusion criteria for data extraction, involving 7187 participants. Of these, 47% received ANP treatment, while 53% were assigned to the placebo group. The baseline characteristics of both groups were similar, as outlined in Table 1. A summary of the findings from each study is presented in Table 2.

### 3.1. Demographics

The average age across the studies varied from 55.3 to 73 years, with a mean of 66.18 years. Male participants were the majority, comprising 66.6% (*n* = 4784) of the total sample, while females made up 32.0% (*n* = 2299). Cardiovascular comorbidities were frequently observed:

- Hypertension: reported in 10 studies, affecting 42.5% to 83.1% of participants.
- Coronary artery disease (CAD): reported in 11 studies, with prevalence ranging from 17.5% to 100%.
- Diabetes mellitus: reported in 10 studies, affecting 5% to 39.1% of participants.
- Congestive heart failure (CHF): reported in five studies, with a prevalence ranging from 20.9% to 99.2%.

Left ventricular ejection fraction (LVEF) was provided in five studies, ranging from 29.4% to 60.4%. When reported, the mean heart rate varied from 83.7 to 98.5 beats per minute. Systolic blood pressure was documented in five studies, ranging from 115.5 to 146.5 mmHg, and diastolic blood pressure was reported in three studies, with a range of 67 to 85.5 mmHg. The studies were conducted in various regions: Japan (nine studies), the USA (two studies), Switzerland (two), Germany (one), and China (one).

### 3.2. Mortality

**Figure 2:**
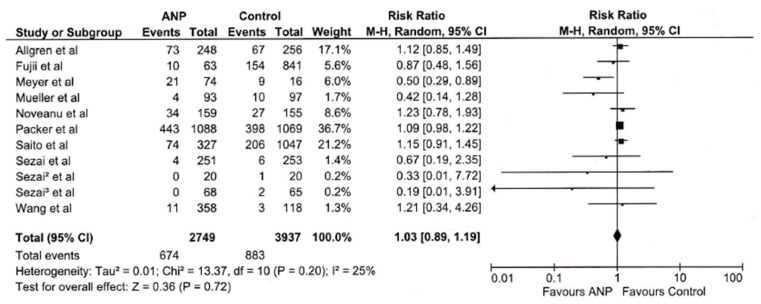
Forest plot showing the effect of ANP on mortality across studies.

The pooled risk ratio is 1.03 (95% CI: 0.89–1.19), indicating no statistically significant difference in mortality risk between ANP and control (*p* = 0.72), as seen in Figure 2. The result is consistent across studies, as shown by the low heterogeneity (I^2^ = 25%, Chi^2^ = 13.37, *p* = 0.20).

### 3.3. Hospital Length of Stay

**Figure 3:**
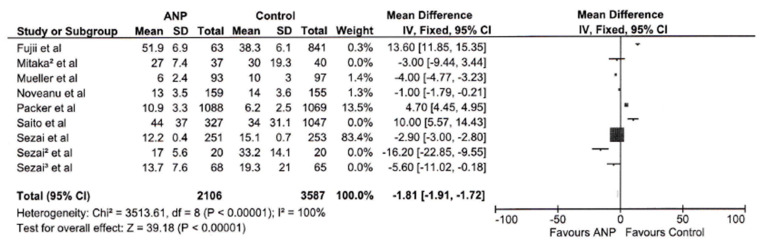
Forest plot displaying hospital stay across studies.

**Figure 4:**
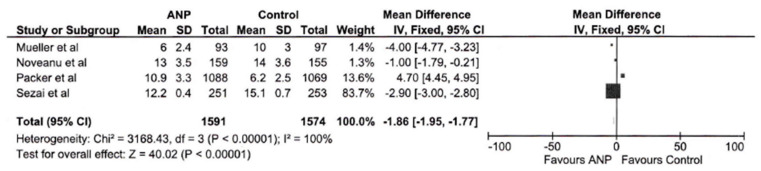
Forest plot displaying subgroup analysis of hospital stay across four studies.

As prespecified in Section 2, subgroup analyses were conducted for hospital length of stay. The mean difference of −1.81 days (95% CI: −1.91 to −1.72) significantly favors ANP (*p* < 0.00001) Figure 3. Due to the very high heterogeneity (I^2^ = 100%, Chi^2^ = 3513.61, *p* < 0.00001), a subgroup analysis involving four studies was conducted. The mean difference of −1.86 days (95% CI: −1.95 to −1.77) still favors ANP (*p* < 0.00001), as seen in Figure 4, but the heterogeneity remains very high (I^2^ = 100%, Chi^2^ = 3168.43, *p* < 0.00001). This suggests that underlying differences in study populations or methodologies were not resolved by subgroup selection.

### 3.4. ICU Length of Stay

As prespecified in Section 2, a subgroup analysis was also performed for ICU length of stay to further investigate the observed heterogeneity. The mean difference +0.13 days (95% CI: 0.00 to 0.25) marginally favors Control (*p* = 0.05) but with high heterogeneity (I^2^ = 96%, Chi^2^ = 159.06, *p* < 0.00001) Supplementary Figure S1. A subgroup analysis showed a mean difference of +0.10 days (95% CI: −0.03 to 0.23), indicating no significant difference between ANP and control (*p* = 0.15), as depicted by Supplementary Figure S2. The subgroup analysis failed to resolve heterogeneity or demonstrate clear treatment superiority.

### 3.5. Serum Creatinine Level

In line with our prespecified plan, a subgroup analysis was conducted for serum creatinine levels across studies that reported this outcome. The mean difference of −0.10 (95% CI: −0.28 to 0.07; *p* = 0.24) in Supplementary Figure S3 shows no significant difference in serum creatinine levels. Due to the high heterogeneity (I^2^ = 98%, Chi^2^ = 394.78, *p* < 0.00001), a subgroup analysis was conducted among five studies. The results showed a mean difference of −0.19 (95% CI: −0.20 to −0.19), *p* < 0.00001 Supplementary Figure S4, significantly favoring ANP. Still, very high heterogeneity (I^2^ = 99%, Chi^2^ = 381.26) suggests caution in interpretation.

### 3.6. Blood Urea Nitrogen Level

The mean difference of +0.37 (95% CI: 0.29 to 0.45; *p* < 0.00001) in Supplementary Figure S5 favors the control group but with high heterogeneity (I^2^ = 97%, Chi^2^ = 114.11). The dominance of one study (Sezai et al. [16]) and high heterogeneity limit the reliability of the pooled estimate.

## 4. Quality Assessment Result

The assessment of randomized controlled trials (RCTs), conducted using the Cochrane risk-of-bias tool (RoB) [29], is summarized in Table 3. Two observational studies were included, with careful matching of patients in the intervention and control groups. The methodology ensures comparability between the groups, allowing any differences observed to be attributed to the intervention under evaluation. Both observational studies achieved scores above seven on the Newcastle–Ottawa Scale (NOS) [30]. The funnel plot depicted in Supplementary Figure S6 reveals bias through its asymmetrical pattern in the distribution of primary outcome studies. This could be due to potential publication bias, the presence of genuine heterogeneity between studies, or possible methodological differences between studies.

## 5. Discussion

This comprehensive meta-analysis, encompassing 15 studies with 7187 participants, yielded fascinating insights into the therapeutic usefulness of ANP in critically ill patients. The findings reveal a complex picture of benefits and limitations that warrant careful consideration in clinical practice.

The analysis demonstrated no statistically significant difference in mortality rates between ANP and control groups (RR 1.03, 95% CI: 0.89–1.19, *p* = 0.72), with relatively low heterogeneity (I^2^ = 25%). In the absence of mortality benefit, this observation implies a somewhat reassuring safety profile. The consistency of this observation across various studies with low heterogeneity strengthens its validity.

A noteworthy positive outcome was the statistically significant reduction in hospital length of stay in patients treated with ANP (MD −1.81 days, 95% CI: −1.91 to −1.72, *p* < 0.00001). This effect persisted in subgroup analyses (MD −1.86 days, *p* < 0.00001), although the marked heterogeneity (I^2^ = 100%) indicates substantial variability in the magnitude of this effect across diverse clinical environments and patient demographics. The reduction in hospital length of stay could bear significant implications for healthcare resource allocation and patient outcomes, albeit the considerable heterogeneity suggests that the effect size may fluctuate considerably across different clinical scenarios.

The influence of ANP therapy on ICU length of stay was less definitive, with initial analyses revealing a marginal disadvantage (MD +0.13 days, *p* = 0.05) that transitioned to non-significance in subgroup analyses (MD +0.10 days, *p* = 0.15). The pronounced heterogeneity (I^2^ = 96%) implies that the effect of ANP on ICU length of stay may be highly contingent on contextual factors that were not captured in the current analysis.

Concerning renal function, the overall analysis of serum creatinine level indicated no significant difference between the groups (MD −0.10, *p* = 0.24). However, subgroup analyses uncovered a significant enhancement in creatinine levels attributable to ANP therapy (MD −0.19, *p* < 0.00001), although the sustained high heterogeneity (I^2^ = 99%) suggests that this effect may vary considerably among different patient populations. Blood urea nitrogen levels were lower in the control group (MD +0.37, *p* < 0.00001), yet the considerable heterogeneity (I^2^ = 97%) and the predominant effect of a singular study constrain the reliability of this finding.

These results should be interpreted within the contextual framework of the study populations, which were predominantly male (66.6%) and exhibited a substantial burden of cardiovascular comorbidities. The geographical distribution of studies, particularly the preponderance of Japanese institutions (9 out of 15 studies), may restrict the generalizability of findings to other demographic groups.

The quality assessment unveiled varying levels of potential bias across studies, with certain studies exhibiting a high risk of selection and performance bias. This variability in study quality, coupled with the high heterogeneity observed in several outcomes, suggests that future research should prioritize more standardized methodologies and rigorously defined patient populations.

While this review focused on placebo-controlled studies to maintain internal validity, future reviews may consider incorporating active-controlled trials to further contextualize the comparative effectiveness of ANP in clinical practice.

## 6. Study Limitations

Several limitations of this meta-analysis warrant acknowledgment. First, the high heterogeneity observed in most outcomes indicates substantial variability in treatment effects across diverse clinical contexts. Secondly, the predominance of studies from a singular geographic region may limit external validity. Finally, the differing quality of the studies included, as indicated by the bias risk assessment, underscores the necessity for caution in the interpretation of certain findings.

Future research directions should include the following:

1. Large-scale, multicenter randomized controlled trials (RCTs) with standardized methodologies to more effectively delineate the most suitable patient populations for the administration of ANP therapy.
2. Studies investigating the cost-effectiveness of ANP treatment, particularly considering the implications of reduced lengths of hospital stay.
3. Explorations into the potential determinants that contribute to the observed variability in treatment outcomes.
4. Research concentrating on distinct patient subgroups to ascertain those individuals who are most likely to derive benefit from ANP therapy.

## 7. Conclusions

This systematic review and meta-analysis provide updated insights into the therapeutic potential of atrial natriuretic peptide (ANP) in critically ill patients. While ANP did not demonstrate a mortality benefit, it consistently reduced hospital length of stay and improved renal function markers in select patient populations. These findings highlight the innovative concept that ANP may exert context-dependent benefits, suggesting its optimal use may depend on careful patient selection rather than broad application across all critically ill individuals. Importantly, this study underscores the necessity for future research to refine dosing strategies, identify biomarkers predictive of response, and better delineate subgroups most likely to benefit. Emphasizing precision medicine in the critical care environment, this work lays the groundwork for reimagining the role of targeted natriuretic peptide therapies to improve outcomes in high-risk patient cohorts.

## Figures and Tables

**Table 1:** Baseline characteristics.

Study	Country	Sample	Age * (yr)	Sex (%)		Admission Etiology	Hypertension (%)	ANP Regimen	CAD (%)	DM (%)	CHF (%)	LVEF (%)	HR/m	Serum Creatinine * (mmol/L)	SBP * (mmHg)	DBP * (mmHg)
				M	F											
Allgren et al., 1997 [14]	USA	504	62 ± 1.0	50.2	49.8	Acute tubular necrosis	58	0.05 → 0.2 µg/kg/min IV over 90 min, then for 24 h	47	29	28	NA	NA	4.6 ± 1.0	127.3 ± 12.3	NA
Mueller et al., 2004 [15]	Switzerland	452	70.8 ± 15.5	57.9	42.1	Acute dyspnea	52	2 µg/kg IV bolus, 0.01 µg/kg/min continuous infusion over 24–48 h	50	23	NA	NA	98 ± 25.5	NA	146.5 ± 29	85.5 ± 19
Sezai et al., 2006 [16]	Japan	40	63.4 ± 12.8	50	50	Cardiac surgery	NA	0.025 µg/kg/min continuous IV infusion for 24 h postoperatively	100	NA	NA	60.2 ± 1.3	NA	NA	NA	NA
Sezai ^2^ et al., 2009 [17]	Japan	504	65.9 ± 0.6	78.9	21.1	Thoracic surgery	70	0.02 μg/kg per minute until patient in ICU	100	44	NA	60.2 ± 1.3	NA	NA	NA	NA
Mitaka et al., 2008 [18]	Japan	40	71.4 ± 8.2	87.5	12.5	Abdominal aortic aneurysm repair	43	0.02 μg/kg/min for 48 h	75	5	NA	NA	NA	NA	NA	NA
Meyer et al., 2009 [19]	Germany	172	64.8 ± 10.8	63.9	36.1	Acute renal failure	40	U5 (5 ng/kg/min), U20 (20 ng/kg/min), U40 (40 ng/kg/min), and U80 (80 ng/kg/min) for 5 days	68	29	21	NA	NA	NA	NA	NA
Sezai ^3^ et al., 2010 [20]	Japan	133	65.9 ± 9.7	85.7	14.3	Coronary artery bypass grafting	74	0.02 μg/kg per minute for 12 h	100	61	NA	NA	NA	NA	NA	NA
Sakamoto et al., 2010 [21]	Japan	10	63.9 ± 15.4	80	20	Pulmonary edema	NA	0.1 μg/kg/min	NA	NA	NA	NA	95.7 ± 16.1	1.05 ± 0.4	NA	NA
Noveanu et al., 2010 [22]	Switzerland	314	69 ± 13	57.6	42.4	Hypoxemic respiratory failure	51	NA	38	NA	27	NA	98.5	NA	127	67
Shibasaki et al., 2015 [23]	Japan	30	63.9 ± 7.9	80	20	Cardiac surgery	53	0.02 μg/kg per minute for 24 h	NA	33	NA	60.4 ± 9.3	NA	0.8 ± 0.3	NA	NA
Wang et al., 2016 [24]	China	476	55.3 ± 13.3	75	25	Acute decompensated heart failure	NA	0.1 µg/kg/min adjusted half hour to 0.15 µg/kg/min for 1 h	27	NA	99	29.4 ± 6.6	83.7 ± 16.9	0.1 ± 0.03	115.5 ± 18.9	NA
Packer et al., 2017 [25]	USA	2157	68.5 ± 11.4	65.8	34.2	Acute heart failure	NA	15 ng/kg/min for 48 h	53	39	3	33.9	86 ± 18.9	1.24 ± 0.36	134.7 ± 17.9	79.2 ± 13.3
Mitaka ^2^ et al., 2017 [26]	Japan	77	73	71.4	28.6	Acute kidney injury associated with cardiac surgery	83	0.02 μg/kg/min until serum creatinine is back to normal	9	24	NA	NA	NA	0.94	NA	NA
Fujii et al., 2018 [27]	Japan	904	66.9	69.5	30.5	Acute kidney injury	51	0.028 μg/kg/min for 2 days or longer	NA	120	NA	NA	NA	NA	NA	NA
Saito et al., 2020 [28]	Japan	1374	68	66.9	33.1	Acute kidney injury	NA	0.019 μg/kg/min for 2 days	NA	NA	NA	NA	NA	0.1	NA	NA

CAD: coronary artery disease; DM: diabetes mellitus; CHF: congestive heart failure; DBP: diastolic blood pressure; HR: heart rate; LVEF: left ventricular ejection fraction; Mitaka^2^: Chieko Mitaka et al. [26].; SBP: systolic blood pressure; Sezai^2^: Akira Sezai, Motomi Shiono, Mitsumasa Hata et al. [17]; Sezai^3^: Akira Sezai, Mitsumasa Hata, Tetsuya Niino et al. [20]; * = mean ± SD.

**Table 2:** Outcomes.

Outcome	Participants	Higgins I^2^	Z Score	*p* Value	Risk Ratio/Mean Difference 95% CI
Mortality	6686	25%	0.36	0.72	1.03 [0.89, 1.19]
Hospital Stay	5693	100%	39.18	<0.00001	−1.81 [−1.91, −1.72]
Hospital Stay Subgroup	3165	100%	40.02	<0.00001	−1.86 [−1.95, −1.77]
ICU Stay	4999	96%	2.00	0.05	0.13 [0.00, 0.25]
ICU Stay Subgroup	3845	98%	1.44	0.15	0.10 [−0.03, 0.23]
Creatinine	3657	98%	1.17	0.24	−0.10 [−0.28, 0.07]
Creatinine Subgroup	3375	99%	86.21	<0.00001	−0.19 [−0.20, −0.19]
Blood Urea Nitrogen	756	97%	9.22	<0.00001	0.37 [0.29, 0.45]

**Table 3:** Cochrane risk-of-bias tool for randomized trials.

Study	Selection Bias	Performance Bias	Detection Bias	Attrition Bias	Reporting Bias
Allgren et al. [14]	Low	Low	Low	Low	Low
Mueller et al. [15]	Low	Unclear	Low	Low	Low
Sezai et al. [16]	Unclear	Low	Low	Low	Low
Sezai^2^ et al. [17]	High	Unclear	Unclear	Low	Low
Mitaka et al. [18]	High	Low	Unclear	Low	Low
Meyer et al. [19]	High	High	High	Low	Low
Sezai^3^ et al. [20]	Low	Low	Unclear	Low	Low
Sakamoto et al. [21]	High	High	Unclear	Low	Low
Noveanu et al. [22]	Low	Low	Low	Low	Low
Shibasaki et al. [23]	Low	Low	Unclear	Low	Low
Wang et al. [24]	Low	Low	Low	Low	Low
Packer et al. [25]	Low	Low	Low	Low	Low
Mitaka^2^ et al. [26]	Low	Low	Unclear	Low	Low

Mitaka^2^: Chieko Mitaka et al. [26]; Sezai^2^: Akira Sezai, Motomi Shiono, Mitsumasa Hata et al. [17]; Sezai^3^: Akira Sezai, Mitsumasa Hata, Tetsuya Niino et al. [20].

## Data Availability

The study data are available from the corresponding author upon reasonable request. Public access to the data is restricted due to privacy and ethical considerations.

## Supplementary Materials

The following supporting information can be downloaded at: https://www.mdpi.com/article/10.3390/jcm14103267/s1, Figure S1: Forest plot displaying ICU length of stay across studies; Figure S2: Forest plot displaying subgroup ICU length of stay across studies; Figure S3: Forest plot displaying serum creatinine levels across studies; Figure S4: Forest plot displaying subgroup serum creatinine levels across studies; Figure S5: Forest plot displaying blood urea nitrogen levels across studies; Figure S6: Funnel Plot.

## Abbreviations

AKI	Acute Kidney Injury
ANP	Atrial Natriuretic Peptide
BNP	Brain Natriuretic Peptide
CAD	Coronary Artery Disease
CHF	Congestive Heart Failure
cGMP	Cyclic Guanosine Monophosphate
CI	Confidence Interval
DBP	Diastolic Blood Pressure
GFR	Glomerular Filtration Rate
HDU	High Dependency Unit
HR	Heart Rate
ICU	Intensive Care Unit
I^2^	Higgins’ Heterogeneity Statistic
LVEF	Left Ventricular Ejection Fraction
MD	Mean Difference
NOS	Newcastle–Ottawa Scale
NT-proBNP	N-terminal pro–Brain Natriuretic Peptide
PMID	PubMed Identifier
PRISMA	Preferred Reporting Items for Systematic Reviews and Meta-Analyses
RCT	Randomized Controlled Trial
RAAS	Renin–Angiotensin–Aldosterone System
RR	Risk Ratio
SBP	Systolic Blood Pressure
SD	Standard Deviation
SOAP	Sepsis Occurrence in Acutely Ill Patients
RoB	Risk of Bias
