# Supplementary material for: Effectiveness of Atrial Natriuretic Peptide in the Treatment of Critically Ill Patients: A Systematic Review and Meta-Analysis
Source: J Clin Med. 2025 May 8;14(10):3267. doi: 10.3390/jcm14103267 (PMC12112224; doi:10.3390/jcm14103267)
Supplement: Supplementary file 1 [file jcm-14-03267-s001.zip › jcm-3572576-supplementary.pdf]

Table of Contents

Figure S1: Forest plot displaying ICU length of stay across studies;
Figure S2: Forest plot displaying subgroup ICU length of stay across studies;
Figure S3: Forest plot displaying serum creatinine lev-els across studies;
Figure S4: Forest plot displaying subgroup serum creatinine levels across studies;
Figure S5: Forest plot displaying blood urea nitrogen levels across studies;
Figure S6: Funnel Plot.

ICU stay

Figure S1

Study or Subgroup	ANP Mean	ANP SD	ANP Total	Control Mean	Control SD	Control Total	Weight	Mean Difference IV, Fixed, 95% CI
Fujii et al	8.9	2.8	63	6.5	2.5	841	3.1%	2.40 [1.69, 3.11]
Mitaka[2] et al	6	2.2	37	6	3	40	1.1%	0.00 [-1.17, 1.17]
Noveanu et al	3	1.7	159	3	1.7	155	10.9%	0.00 [-0.38, 0.38]
Packer et al	2.9	1.7	1088	3	1.7	1069	75.1%	-0.10 [-0.24, 0.04]
Saito et al	5.5	4.4	327	2.8	2.8	1047	6.0%	2.70 [2.19, 3.21]
Sezai[2] et al	5.1	2.1	20	6.3	1.6	20	1.2%	-1.20 [-2.36, -0.04]
Sezai[3] et al	3.6	1.9	68	4.4	2.6	65	2.6%	-0.80 [-1.58, -0.02]
**Total (95% CI)**			**1762**			**3237**	**100.0%**	**0.13 [0.00, 0.25]**

Heterogeneity: $Chi^2 = 159.06$, $df = 6$ ($P < 0.00001$); $I^2 = 96\%$
Test for overall effect: $Z = 2.00$ ($P = 0.05$)

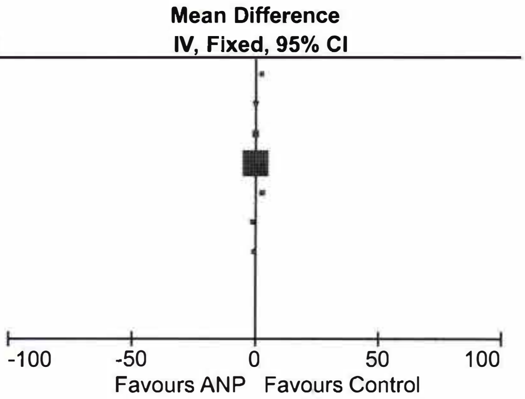

Figure S2

	ANP			Control				Mean Difference
**Study or Subgroup**	**Mean**	**SD**	**Total**	**Mean**	**SD**	**Total**	**Weight**	**IV, Fixed, 95% CI**
Noveanu et al	3	1.7	159	3	1.7	155	11.9%	0.00 [-0.38, 0.38]
Packer et al	2.9	1.7	1088	3	1.7	1069	81.6%	-0.10 [-0.24, 0.04]
Saito et al	5.5	4.4	327	2.8	2.8	1047	6.6%	2.70 [2.19, 3.21]
**Total (95% CI)**			**1574**			**2271**	**100.0%**	**0.10 [-0.03, 0.23]**

Heterogeneity: Chi² = 109.09, df = 2 (P < 0.00001); I² = 98%

Test for overall effect: Z = 1.44 (P = 0.15)

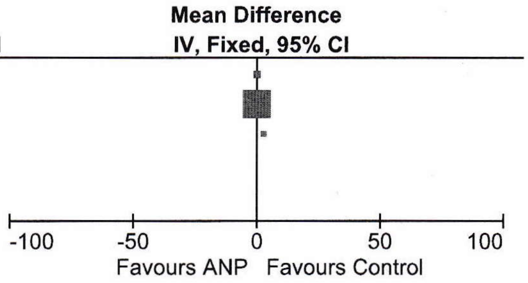

creatinine

Figure S3

	ANP			Control				Mean Difference
**Study or Subgroup**	**Mean**	**SD**	**Total**	**Mean**	**SD**	**Total**	**Weight**	**IV, Random, 95% CI**
Allgren et al	2.8	1.7	248	3	1.7	256	11.8%	-0.20 [-0.50, 0.10]
Meyer et al	3.2	1.3	114	2.82	1.1	58	9.9%	0.38 [0.01, 0.75]
Mitaka et al	0.9	0.2	20	1.5	1.1	20	7.4%	-0.60 [-1.09, -0.11]
Mitaka[2] et al	1.2	0.5	37	1.2	0.6	40	13.2%	0.00 [-0.25, 0.25]
Packer et al	1.4	0.3	1088	1.3	0.4	1069	17.9%	0.10 [0.07, 0.13]
Sezai et al	0.9	0.02	251	1.1	0.03	253	18.0%	-0.20 [-0.20, -0.20]
Sezai[2] et al	1.8	1.2	20	2.6	1.8	20	2.8%	-0.80 [-1.75, 0.15]
Sezai[3] et al	1.1	0.4	68	1.3	0.9	65	13.4%	-0.20 [-0.44, 0.04]
Shibasaki et al	0.7	0.8	15	0.8	0.9	15	5.6%	-0.10 [-0.71, 0.51]
**Total (95% CI)**			**1861**			**1796**	**100.0%**	**-0.10 [-0.28, 0.07]**

Heterogeneity: $Tau^2 = 0.04$; $Chi^2 = 394.78$, df = 8 ($P < 0.00001$); $I^2 = 98\%$
Test for overall effect: Z = 1.17 (P = 0.24)

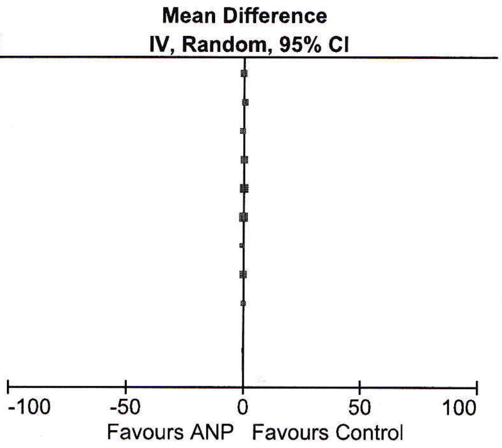

Creatinine subgroup

Figure S4

Study or Subgroup	ANP Mean	ANP SD	ANP Total	Control Mean	Control SD	Control Total	Weight	Mean Difference IV, Fixed, 95% CI
Allgren et al	2.8	1.7	248	3	1.7	256	0.0%	-0.20 [-0.50, 0.10]
Mitaka[2] et al	1.2	0.5	37	1.2	0.6	40	0.0%	0.00 [-0.25, 0.25]
Packer et al	1.4	0.3	1088	1.3	0.4	1069	2.2%	0.10 [0.07, 0.13]
Sezai et al	0.9	0.02	251	1.1	0.03	253	97.7%	-0.20 [-0.20, -0.20]
Sezai[3] et al	1.1	0.4	68	1.3	0.9	65	0.0%	-0.20 [-0.44, 0.04]
**Total (95% CI)**			**1692**			**1683**	**100.0%**	**-0.19 [-0.20, -0.19]**

Heterogeneity: $Chi^2 = 381.26$, df = 4 ($P < 0.00001$); $I^2 = 99\%$

Test for overall effect: Z = 86.21 ($P < 0.00001$)

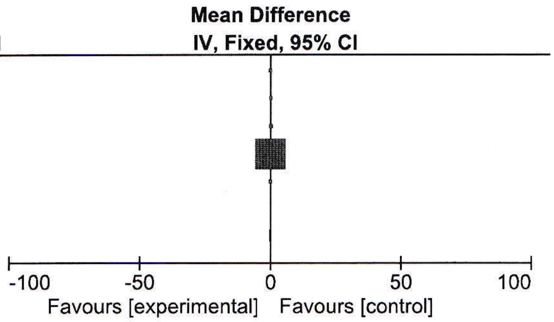

BUN

Figure S5

Study or Subgroup	ANP Mean	ANP SD	ANP Total	Control Mean	Control SD	Control Total	Weight	Mean Difference IV, Fixed, 95% CI
Meyer et al	49.8	27.2	114	49.9	24.1	58	0.0%	-0.10 [-8.06, 7.86]
Mitaka et al	11	2.2	20	17	1.6	20	0.4%	-6.00 [-7.19, -4.81]
Sezai et al	18.9	0.5	251	18.5	0.4	253	99.5%	0.40 [0.32, 0.48]
Sezai² et al	38.8	11.9	20	50.7	25.2	20	0.0%	-11.90 [-24.11, 0.31]
**Total (95% CI)**			**405**			**351**	**100.0%**	**0.37 [0.29, 0.45]**

Heterogeneity: $Chi^2 = 114.11$, $df = 3$ ($P < 0.00001$); $I^2 = 97\%$
Test for overall effect: $Z = 9.22$ ($P < 0.00001$)

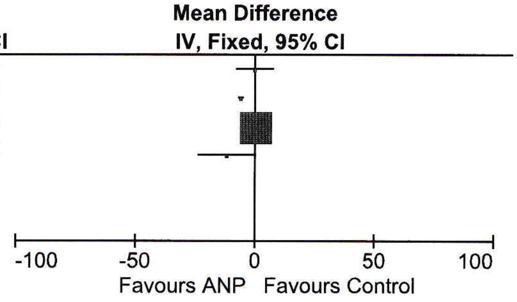

Figure S6

Funnel Plot of Mean Differences

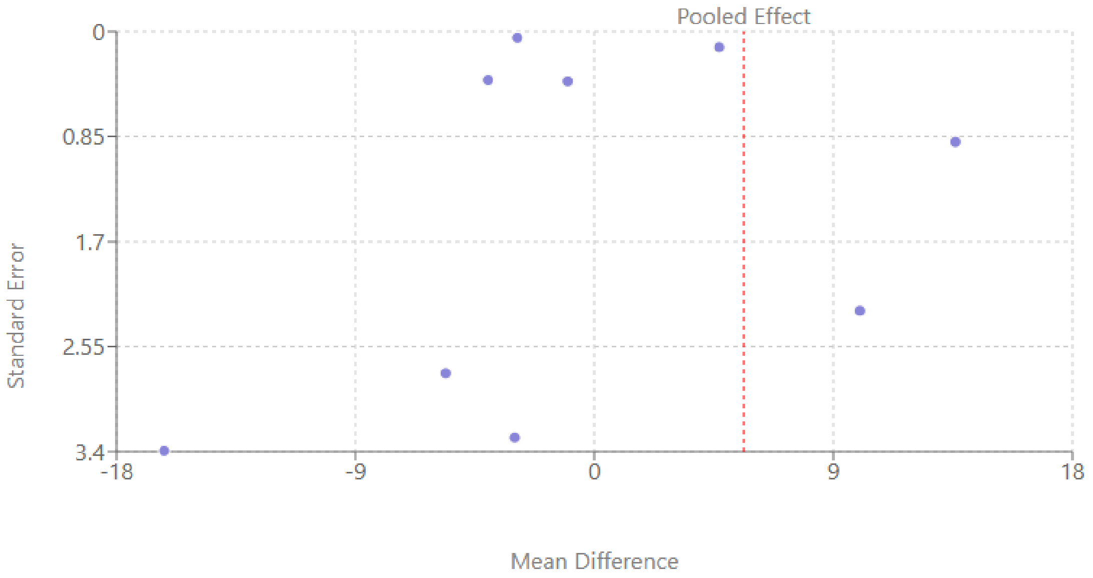

This funnel plot displays mean differences (x-axis) against standard error (y-axis, reversed). Each point represents a study, with the size reflecting the study's sample size. The red dashed line indicates the pooled effect size. Asymmetry in the plot may suggest publication bias or other systematic differences between studies.
